# Cellular Redox State Acts as Switch to Determine the Direction of NNT-Catalyzed Reaction in Cystic Fibrosis Cells

**DOI:** 10.3390/ijms22020967

**Published:** 2021-01-19

**Authors:** Maria Favia, Anna Atlante

**Affiliations:** 1 Istituto di Biomembrane, Bioenergetica e Biotecnologie Molecolari (IBIOM)–CNR, Via G. Amendola 122/O, 70126 Bari, Italy; 2 Dipartimento di Bioscienze, Biotecnologie e Biofarmaceutica, Università di Bari, Via E. Orabona 4, 70126 Bari, Italy

**Keywords:** nicotinamide nucleotide transhydrogenase, cystic fibrosis, mitochondria, glucose-6-phosphate dehydrogenase, NADPH, antioxidant

## Abstract

The redox states of NAD and NADP are linked to each other in the mitochondria thanks to the enzyme nicotinamide nucleotide transhydrogenase (NNT) which, by utilizing the mitochondrial membrane potential (mΔΨ), catalyzes the transfer of redox potential between these two coenzymes, reducing one at the expense of the oxidation of the other. In order to define NNT reaction direction in CF cells, NNT activity under different redox states of cell has been investigated. Using spectrophotometric and western blotting techniques, the presence, abundance and activity level of NNT were determined. In parallel, the levels of NADPH and NADH as well as of mitochondrial and cellular ROS were also quantified. CF cells showed a 70% increase in protein expression compared to the Wt sample; however, regarding NNT activity, it was surprisingly lower in CF cells than healthy cells (about 30%). The cellular redox state, together with the low mΔΨ, pushes to drive NNT reverse reaction, at the expense of its antioxidant potential, thus consuming NADPH to support NADH production. At the same time, the reduced NNT activity prevents the NADH, produced by the reaction, from causing an explosion of ROS by the damaged respiratory chain, in accordance with the reduced level of mitochondrial ROS in NNT-loss cells. This new information on cellular bioenergetics represents an important building block for further understanding the molecular mechanisms responsible for cellular dysfunction in cystic fibrosis.

## 1. Introduction

Nicotinamide adenine dinucleotide (NAD) and nicotinamide adenine dinucleotide phosphate (NADP) act as “electron carriers”. Despite their structural similarity, these molecules—in their reduced forms, NADH and NADPH—are required to drive different and specific cellular processes [1,2,3]. In fact, if NAD, accepting electrons in catabolic pathways, requires its pool to be maintained in an oxidized state, in contrast, maintenance of the nuclear and mitochondrial genomes, cell signaling, antioxidative defense, redox balance and all anabolic reactions require the reductive power stored within NADPH [4,5]. An arsenal of NADPH-regenerating enzymes (NRE) identified in various metabolic pathways—among them Glucose-6-phosphate dehydrogenase (G6PDH), malic enzyme and isocitrate dehydrogenase—ensures that this pyridine nucleotide remains in its reduced form. These enzymes are the main contributors of cellular NADPH pool [6] so much so that an increase in the expression of lipid synthesis or antioxidant defense genes is associated with the induction of them [7,8,9], as well as the loss of one NADPH-producing enzyme can be compensated by the induction of another [7].

The complexity of NADPH homeostasis regulation in eukaryotic systems is essentially due to membranes being impermeable to NADPH. In this regard, it should be borne in mind that neither NAD(H) nor NADP(H) are transported across intracellular membranes [10,11], and multistep shuttles involving compartmentalized redox reactions are used to transfer electrons between the mitochondria and cytosol [12]. This organization facilitates the maintenance of different NADH/NAD and NADPH/NADP+ ratios in each subcellular location and allows for the execution of compartment-specific metabolic processes.

Interestingly, the redox states of NAD and NADP are linked to each other in the mitochondria of many organisms since the enzyme nicotinamide nucleotide transhydrogenase (NNT), an integral protein of the inner mitochondrial membrane [13], acting by utilizing the mitochondrial membrane potential (mΔΨ), catalyzes the transfer of redox potential between these two coenzymes, reducing one at the expense of the oxidation of the other [10,14].

In vivo the formation of NADPH by NNT, at the expense of NADH oxidation, is highly favored. Hence NADH, besides to be a source of fuel for the electron transport system to generate the mΔΨ needed to support NNT activity [15], is also a substrate for NNT. It also appears that NNT is the major mitochondrial source of NADPH, contributing 45% of the total NADPH supply [16,17,18], so as to be considered a key antioxidant enzyme. However, under certain pathophysiological conditions, NNT reaction may change direction, i.e., to operate in reverse: this occurs at the expense of NADPH, thereby disrupting antioxidant defense [17,19,20].

Here, we show that the metabolic demand associated with the ΔF508 mutation reverses the direction of the NNT reaction, generating NADH from NADPH and NAD^+^, resulting in the antioxidant defense loss and metabolic adaption.

## 2. Results

During infections associated to CF, a high NADPH level is needed due to augmented oxidative stress [21,22,23,24].

Recently, we found that CF cells have increased NADPH oxidase (NOX) activity and decreased glutathione reductase (GR) activity, both NADPH consuming enzymes (NCE), but with opposite role: if NOX utilizes NADPH to produce ROS, NADPH serves to recover the GSH level from GSSG in the GR reaction. Furthermore, we proved that both defective CFTR and NOX/GR activity imbalance contribute to NADPH and GSH level decrease and ROS overproduction in CF cells. In parallel, we detected low NADPH level [24,25]—likely due to lower G6PDH activity—and high NADH level, as expected, being its oxidation impaired since ETC activity decreased in CF [22,23,24,25].

Considering that the balance between the consumption of NADPH via NOX [25], and the regeneration of NADPH by NADPH regenerating enzymes, e.g., G6PDH, within the cytosol, may overall depend on metabolic/redox state of the cell, we suggest that NNT, enzyme providing a link between the NADP/NADPH and NAD/NADH pools and the proton motive force, may have a very important role for redox metabolic regulation in CF.

### 2.1. NNT Expression and Activity in CF Airway Cells

**Figure 1:**
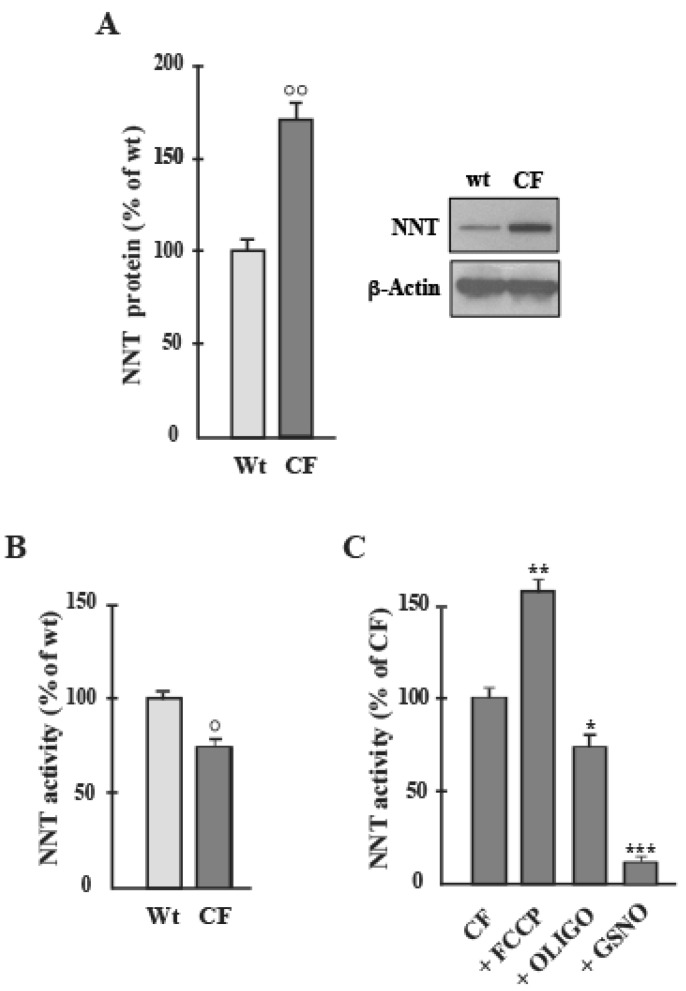
NNT protein level and activity in airway cells. (**A**) NNT protein expression in Wt and CF cells was detected by Western blot analyses. The blot incubated for each antibody was stripped and reprobed with anti ß-actin antibody. Protein amount was expressed, after normalization with respect to corresponding β-actin, as a percentage of the content inWt cells to which a value of 100 was CCCP (3 μmol/L), OLIGO (1.5 ng/ml) and GSNO (1 mmol/L) were added 15 min before activity determination. Values were subjected to statistical analysis (Wt, *n* = 4; CF, *n* = 5): (**B**), ° *p* < 0.05 when comparing CF with the Wt-samples); (**C**), * *p* < 0.05, ** *p* < 0.01, *** *p* < 0.001 when comparing untreated cells with the cells in the presence of compounds).

In order to find out whether NNT expression and activity were altered in CF and, at the same time, to detect the direction of the reaction, we analyzed these characteristics in human bronchial epithelial cells using western blotting and spectrophotometry. Once the presence of NNT was ascertained, we measured the protein level: CF cells showed a 70% increase in protein expression compared to the Wt sample (Figure 1A).

Regarding NNT activity, it is to consider that NNT is the only enzyme to act coupling production of NADPH to the rate of aerobic respiration. Comparing CF and Wt samples, NNT activity was surprisingly lower in CF cells than healthy cells (about 30%), with activity rates of 52.6 ± 1.2 in the Control and 36.5 ± 0.5 nmol/min/mg cell protein in the CF group (Figure 1B), values in line with Sheeran et al. [6] data in human heart mitochondria.

In order to confirm that the effect was specific for NNT activity, S-nitrosoglutathione (GSNO, 1 mM), a strong inhibitor of NNT, was found to cause a collapse of the activity (Figure 1C) [6].

Further, considering that the NNT reaction is strictly dependent on mΔΨ, it was verified that NNT activity responds to changes in the proton motive force. Confirmation of this was obtained from the observation that Carbonyl cyanide m-chlorophenyl hydrazone (CCCP, 3 μM), the proton-gradient uncoupler (Figure 1C), and Oligomycin (OLIGO, 1.5 ng/mL), ATP synthase inhibitor, caused a stimulation (about 25%) and a decrease (15%), respectively, in NNT activity (Figure 1C), in accordance with [13].

Now, to disclose how NNT works under this specific pathological condition and how it participates to the redox homeostasis, we quantified the levels of NADPH and NADH in CF cells under different mitochondrial respiratory state, either in the absence or presence of NNT inhibitor.

**Figure 2:**
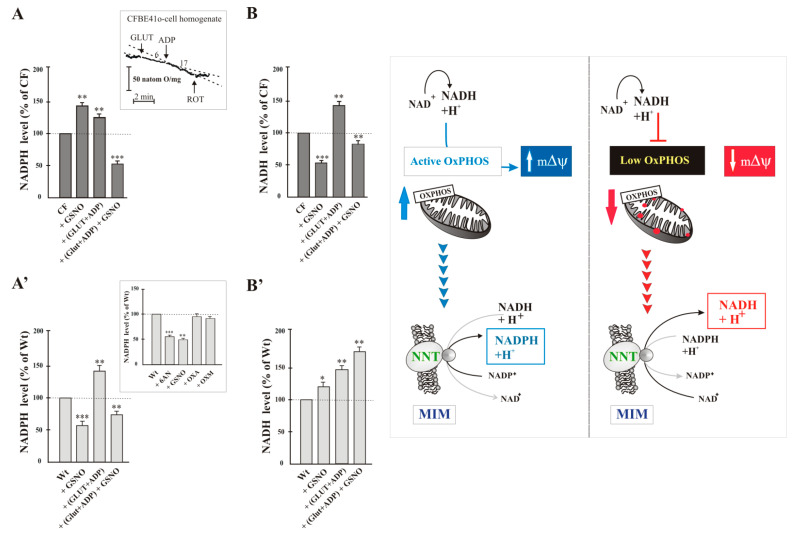
NADPH and NADH levels in airway cells. The amounts of NADPH (**A**-**A’**) and NADH (**B**-**B’**) were assayed as in [24,25]. Cells were incubated for 15 min in the absence or presence of GSNO (1 mmol/L), GLUT (10 mmol/L), ADP (1 mmol/L). Each level value was expressed as % of control, i.e., sample in the absence of compound, to which value 100 was given. Values were subjected to statistical analysis (Wt, *n* = 5; CF, *n* = 5): * *p* < 0.05; ** *p* < 0.01; *** *p* < 0.001 when comparing untreated cells with the cells in the presence of compounds. Inset to (**A**) functionality of mitochondria verified by the ability of (GLUT + ADP) to stimulate oxygen consumption. Inset to (**A’**) NADPH level measured in Wt cells treated with 6AN (5 mmol/L), GSNO (1 mmol/L), OXA (1 mmol/L), OXM (3 mmol/L).

Before proceeding in this sense, we checked that:

(i) first, lower level of NADPH and higher level of NADH were confirmed in CF than in Wt cells (not shown), as in [25];
(ii) the cellular enzymes most involved in the production of NADPH are the cytosolic enzyme G6PDH and the mitochondrial NNT; neither Malic enzyme, nor isocitrate dehydrogenase contributed substantially to the production of NADPH in the cell. To test this, the inhibitors of NADP-dependent enzymes were added separately to cells and after 10 min NADPH level was assayed and compared to that of control cells treated with vehicle only. NADPH level did not change significantly in Wt cells treated with oxalate (OXA, 1 mM), a specific inhibitor of malic enzyme [26], or oxalomalate (OXM, 3 mM), a competitive inhibitor of NADP+-dependent isocitrate dehydrogenase [27], but it was markedly reduced by 6-aminonicotinamide (6AN, 5 mM, by approximately 50%), compound that inhibit G6PDH [24], and by GSNO (1 mM, by approximately 45%) (see inset to Figure 2A’).

When GSNO was added to CF cells (No-NNT cells), we surprisingly observed much higher NADPH level (45%) than that of CF cells not treated with GSNO (Figure 2A); on the contrary after the same treatment NADH level was lower (45%) in No-NNT cells than in untreated cells (Figure 2B). Already these data led us to suspect that NNT in CF cells works in the reverse direction. To confirm this, we measured the levels of NADH and NADPH under active mitochondrial respiratory state condition. In the presence of Glutamate plus ADP (GLUT + ADP), the citric acid cycle (TCA) and electron transport chain (ETC) can actively both generate NADH and maintain the proton (H+) gradient, optimal conditions which might to allowing for NNT to create NADPH. After we verified the residual functionality of mitochondria by the ability of glutamate (10 mM) and ADP (1 mM) to stimulate oxygen consumption and thus respiratory activity (see inset to Figure 2A), we quantified the levels of reduced pyridine dinucleotides. (GLUT + ADP) treatment increased NADPH level by 25% in CF cells, with respect to untreated CF cells, and it was strongly sensitive to GSNO which caused a strong reduction of NADPH content (Figure 2A), this confirming that NNT operated in the forward direction, that is it had produced NADPH by likely utilizing NADH, provided by the GLUT + ADP couple, and mΔΨ.

Therefore, the increase in the level of NADPH observed in Figure 2A, i.e., in the presence of GSNO or (GLUT + ADP), is due to different conditions (see Scheme to Figure 2): in the first case, NADPH accumulates since GSNO prevents NNT to operate in reverse direction; while, in the second, the increase is likely due to NADPH formation due to the forward reaction of NNT in the presence of GLUT + ADP.

As expected, (GLUT + ADP) treatment increased also NADH level (42%) (Figure 2B).

These findings proposed that NNT may catalyze both reactions, i.e., reverse and forward, to generate NADH and NADPH respectively, depending on cellular redox state. The situation is completely different in Wt cells where NADPH level strongly dropped in the presence of GSNO (Figure 2A’). It increased upon (GLUT + ADP) addition to cells, lowering again in presence of GSNO. Regarding NADH, its level increased in the presence of GSNO, even more under active respiratory state, both in the absence and, more much, presence of GSNO (Figure 2B’).

Data suggest that in CF cells NNT operates in a reverse mode to generate NADH from NADPH and NAD+, resulting in reductive stress and metabolic adaption. NADH production fuels mitochondrial OXPHOS, which combined with the compromised antioxidant capacity of NADPH likely results in mitochondrial ROS production increase (see below) and oxidative damage (see inset to Figure 2).

### 2.2. Interdependence of NADPH-Linked Enzymes, i.e., G6PDH and NNT

Now, considering the close association between low NADPH and reduced G6PDH activity occurring in CF cells, where NOX-dependent ROS level increased [23,24,25], in order to verify the interdependence of cytosolic G6PDH—essential enzyme for protection against cellular ROS—and NNT—whose reaction direction is strictly dependent upon cell redox state—the NNT activity was studied in the presence of 6AN, G6PDH inhibitor, and, vice versa, the activity of G6PDH in presence of GSNO.

**Figure 3:**
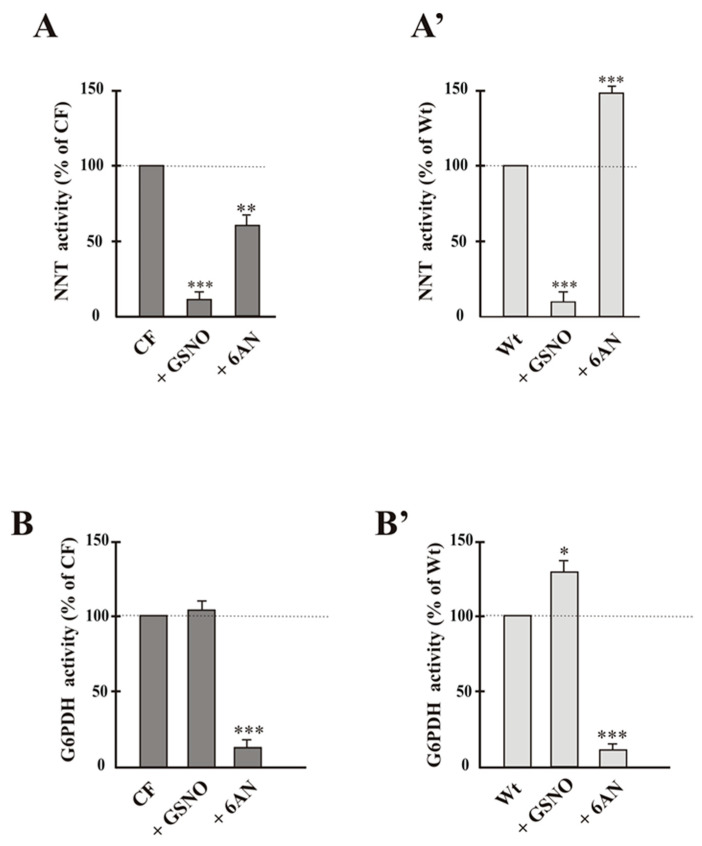
NNT (**A**-**A’**) and G6PDH (**B**-**B’**) activities in the presence of own and the specific inhibitor of the other enzyme. Values were subjected to statistical analysis (Wt, *n* = 5; CF, *n* = 4). Each value was expressed as % of control, i.e., sample in the absence of compound, to which value 100 was given. * *p* < 0.05; ** *p* < 0.01; *** *p* < 0.001 when comparing untreated cells with the cells in the presence of compounds, i.e., 6AN (5 mmol/L), GSNO (1 mmol/L).

NNT activity, collapsed by GSNO, lowered in the presence of 6AN (Figure 3A), thus suggesting that if G6PDH does not work, less NADPH is available for NNT reaction in CF, although the enzymes belong to different compartments. Conversely GSNO was without effect on G6PDH activity (Figure 3B).

Regarding Wt cells, where NNT reaction direction is forward, the pattern of both enzyme activities showed that the tested inhibitors were able to interfere with both metabolic pathways (Figure 3A’,B’): activities resulted mutually increased in the presence of the specific inhibitor of the other enzyme. In fact, NNT activity increases (35%) in the presence of 6AN (Figure 3A’), unlike what happens in CF (Figure 3A); similarly, G6PDH increased in the presence of GSNO (Figure 3B’).

### 2.3. NNT Activity Regulates Mitochondria-Dependent ROS Production

Having previously established that the reduced ROS level by 6AN in CF cells was likely due to the reduced level of NADPH [23,24], product of G6PDH and substrate of NOX [25], in order to investigate if mitochondrial enzyme NNT, involved in modulating the levels of the reduced pyridine dinucleotides, is implicated in the excessive synthesis of mitochondrial ROS then modulating their production, GSNO effect on ROS level was investigated.

**Figure 4:**
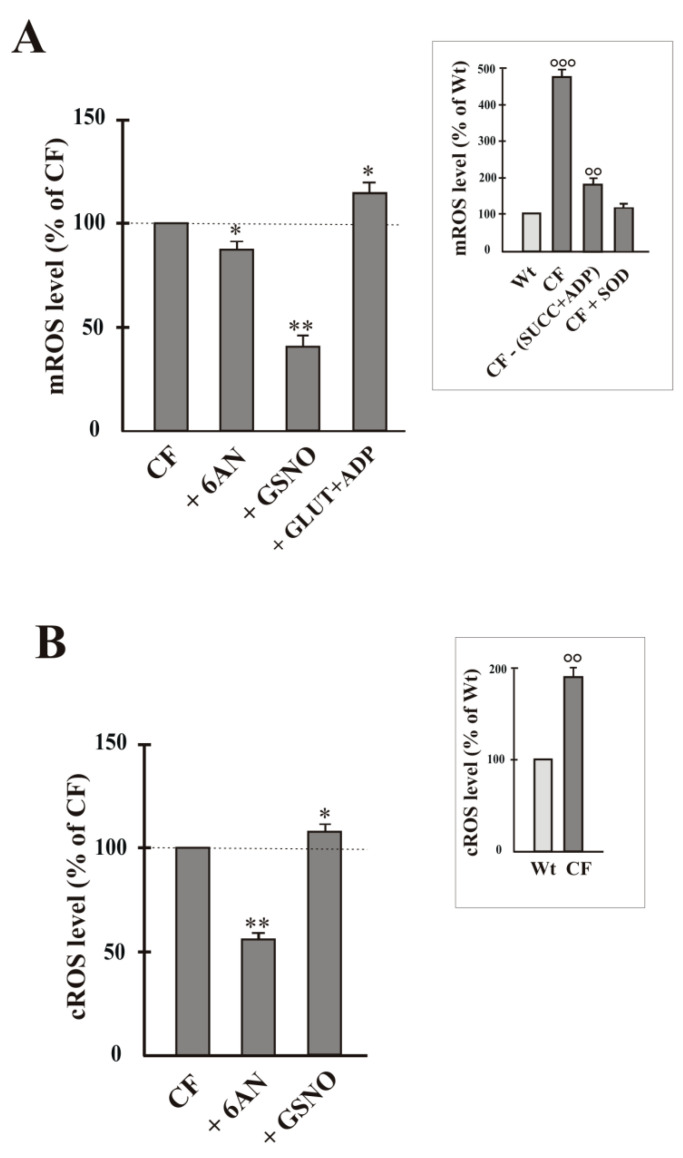
ROS level in airway cells. Mitochondrial and cytosolic O_2_^−•^ production was detected by using the MitoSox dye (**A**) and according to the adrenochrome method (**B**), respectively (see Method Section). The O_2_^−•^ level value was expressed as % of control, i.e., sample in the absence of compound, to which value 100 was given. Cells were incubated for 15 min in the absence or presence of 6AN (5 mmol/L), GSNO (1 mmol/L), GLUT (10 mmol/L) + ADP (1 mmol/L). Values were subjected to statistical analysis (for both Wt and CF, *n* = 4). * *p* < 0.05; ** *p* < 0.01 when comparing untreated CF cells with the same cells in the presence of compounds. In the Insets, ROS level value was expressed as % of control (Wt) to which value 100 was given. °° *p* < 0.01; °°° *p* < 0.001 versus Wt.

Regarding mitochondrial ROS, we previously demonstrated that the mitochondrial O_2_^−•^ overproduction is borne by Complex I [2]. In Figure 4A a summary of four experiments is shown where emission fluorescence (580 nm) value, obtained when Mitosox (1 μM) was added to the cell homogenate containing respiring mitochondria, is reported. This emission fluorescence value was really due to the mitochondrial O_2_^−•^-dependent MitoSOX oxidation. In accordance with Atlante et al. [22], mitochondrial O_2_^−•^ production was about 5-fold higher in CF homogenate with respect to Wt (see inset to Figure 4A). Further, as a control to confirm the validity of the Mitosox procedure to detect mitochondrial ROS, no significant MitoSox oxidation occurred when CF homogenate was incubated in the absence of SUCC + ADP, as well as it was almost completely reduced at values almost equal to the control values in the presence of the antioxidant superoxide dismutase (SOD, 5 units/mL) (see inset to Figure 4A). For their part, GSNO caused a strong decrease of mROS level and 6AN a slight, but significant, decrease MitoSOX oxidation in CF (Figure 4A). When mitochondria were energized by (GLUT + ADP) addition, i.e., the experimental condition which restores the forward direction of the reaction catalyzed by NNT, as observed in Figure 2B, ROS level increased.

By using epinephrine as ROS detecting method, we first confirmed that O_2_^−•^ level doubled in CF cells (see inset to Figure 4B) and, in turn, it is almost halved in the presence of 6AN, according to [22]. Cytosolic ROS level lightly increased in the presence of GSNO with respect to CF control (Figure 4B).

Regarding Wt cells, we confirmed, according to [24], that 6AN caused a 30% increase, while GSNO was without effect on level of ROS of cytosolic origin (not shown). About ROS of mitochondrial origin, both 6AN and GSNO were without effect (not shown).

## 3. Discussion

NNT, significant producer of mitochondrial NADPH [16,17,18], is considered a key antioxidant enzyme in the cell [28], therefore its dysfunction compromises the ability of mitochondria to deal with oxidative stress [28]. Consistently, it was shown that H295R cells, whit NNT stably knocked down, undergo oxidative stress [29]. Similarly, NNT ablation caused defects in energy metabolism in other mouse tissues (heart, liver, pancreas).

Overwhelming oxidative stress is a critical step for CF disease progression [23]. Previously, we described how NRE, i.e., G6PDH, and NCE, i.e., NOX and GR, participate in modulating intracellular GSH level and in the fight against oxidative stress in CF. We also proved that G6PDH-depending NADPH is preferentially channeled towards NOX, rather than GR [25]. In this complex scenario, NNT could have a very important role in regulating the balance between NADH/NAD+ and NADPH/NADP+ ratios, with consequences for metabolic regulation of entire cell.

### 3.1. How NNT Works in CF Cells

Here we demonstrate for the first time that, despite higher NNT protein level (70%) (Figure 1A), there is a significant decrease (30%) in NNT activity in CF than WT cells (Figure 1B). This discrepancy is not surprising. After all, by existing a direct relationship between NADPH level and mΔΨ, as suggested by Aon and colleagues [30], and given that the activity of several OXPHOS proteins are markedly impaired in CF [22,24], NNT may become at the same time either the target or the main responsible of the remodeling of the cytosolic redox status, whose unbalancing depends on many factors including mitochondria.

One could assume that the higher NNT protein level in CF (Figure 1A) reflects needs of the cell that is getting ready to counteract the looming oxidative stress. However, only its functional product, i.e., the activity of NNT, gives us an idea of the actual metabolic state the CF cell is really in, taking into account the cellular dynamics involving the different cellular compartments which communicate with each other and where the metabolic pathways take place.

Therefore, in light of this data (Figure 1A,B), we set out to understand the direction of the NNT reaction in CF cells. In his fascinating work, Nickel et al. [17] proposed that this enzyme has a dual role serving either pro- or anti-oxidative processes depending on the metabolic state of a cell. By measuring the levels of reduced adenindinucleotides, i.e., NADPH and NADH, under different cell energetic states, we have obtained the tangible evidence that this is true also in CF cells.

NADPH increase as well as NADH decrease, observed in the presence of NNT inhibitor GSNO (Figure 2A,B), strongly support the hypothesis that NNT works in reverse mode under the condition the CF cell is in [22]; then NADH is exclusively preserved for OXPHOS (Figure 2B), neglecting the antioxidative defense. Contrarily, under an excess of NADH, i.e., when (GLUT + ADP) are added to homogenate, NNT changes direction, i.e., it acts to transfer hydride ion to generate NADPH (Figure 2A), thus indirectly helping to remove any ROS actively generated by respiring mitochondria.

Different is the situation concerning WT cells: NNT works always forward. It utilizes NADH to produce NADPH, whose level is strongly reduced in the presence of GSNO, but further increased when mitochondria are energized (Figure 2A’). About NADH, its level progressively increases as observed in Figure 2B’. This is in agreement with previous findings in which silencing of NNT expression increases NADH/NAD+ ratio in osteosarcoma cells [31], although it seems that factors, like cell type, differentiation state, and the compensatory effect by other redox-metabolizing enzymes, may affect the outcome of NNT loss.

### 3.2. Crosstalk between the Cytosolic and Mitochondrial NADPH Pools

**Figure 5:**
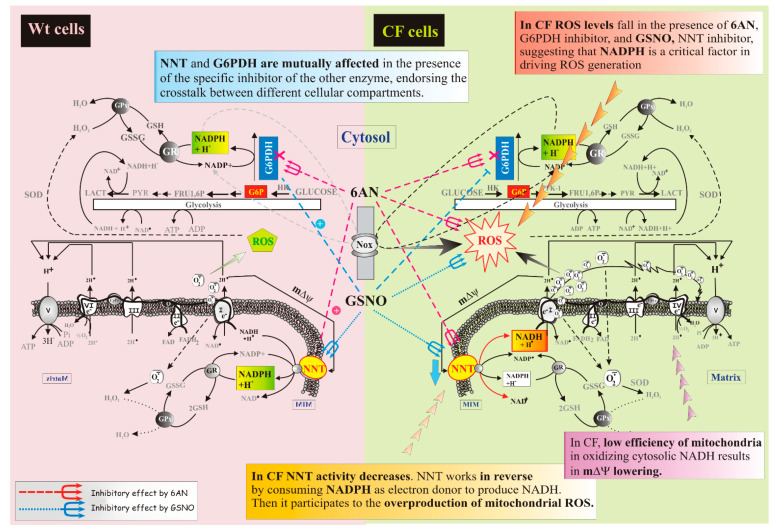
Representation of the cellular energy state. Comparison between Wt and CF conditions: different mode of action of NNT protein. Arrows are defined in the figure, bottom left.

Despite the requirements of the compartmentation, several considerations, arising from the data of Figure 3, suggest that G6PDH and NNT, despite their different cellular localization, strictly dialogue, thus participating to maintenance of cell redox state, thanks to the fact that nucleotides have different metabolic roles in the different compartments (see Figure 5).

In particular, while in Wt cells each of them, i.e., G6PDH and NNT, can take charge of NADPH supply in case that its depletion is due to the loss of the other enzyme, consistent with Figure 3A’,B’, as regards CF cells, where NNT works in reverse, the situation is different., NNT activity decreases in the presence of 6AN, responsible of G6PDH-dependent NADPH impoverishment; whereas GSNO is without effect on G6PDH. These data suggest a crosstalk existing between the cytosolic and mitochondrial NADPH pools, strictly dependent on enzymatic activities as well as cellular redox state.

### 3.3. How ROS Levels Respond to NNT Loss

In a broad cellular context, the different cellular compartments, i.e., cytosol and mitochondria, are mutually informed of what the other compartment needs in order to function at their best. Hence, in this context, the implications of reduced NNT activity by GSNO on ROS originating in the two compartments were investigated.

As shown in Figure 4A, MitoSox oxidation, i.e., mitochondrial ROS level, was reduced in the presence of GSNO; slight, but significant, reduction was also in the presence of 6AN.

When the cytosolic ROS level was detected, the response to inhibitors, i.e., GSNO and 6AN, was different: a slight increase of ROS in the presence of GSNO and a halving of ROS in the presence of 6AN were observed (see Figure 4B).

## 4. Materials and Methods

### 4.1. Reagents

All enzymes and biochemicals were purchased from Sigma Chemical Co. (St Louis, MO, USA).

### 4.2. Cell Culture

Experiments were performed with two human bronchial epithelial cell lines: CFBE41o-cells overexpressing F508del CFTR (CFBE), and respective control, i.e., CFBE41o-cells stably expressing wildtype CFTR (Wt-CFBE), referred to as “CF cells” and “Wt cells” in the text, respectively.

For cell growth conditions and protein content measurement, see [22].

### 4.3. Cell Suspension and Homogenate Preparation

Cell suspension and homogenate preparation were obtained as reported in [24]. Briefly, plated cells were repeatedly washed with phosphate-buffered saline (PBS), scraped and collected to obtain the suspension. Cell homogenate was obtained by breaking up cell suspension by about 10 strokes with a Dounce homogenizer at room temperature.

### 4.4. Protein Extraction and Western Blotting

Confluent cells were lysed essentially as in [32]. An aliquot of 30 μg of protein was separated by 4–15% SDS-PAGE Criterion TGX precast gel (Bio-Rad Laboratories, Inc., Philadelphia, PA, USA) and blotted for NNT protein by using polyclonal NNT antibody (dilution 1:400; Sigma Chemical Co., St Louis, MO, USA) or ß-actin by using monoclonal antibody (dilution 1:5000; Sigma Chemical Co., St Louis, MO, USA). The secondary antibodies were anti-mouse IgG for monoclonal antibody and anti-rabbit IgG for polyclonal antibody (Sigma). Immunocomplexes were detected with LumiGlo reagent (Cell Signalling, Danvers, MA) and densitometric quantification and image processing were carried out using Adobe Photoshop and the Image software package (version 1.61, National Institutes of Health, Bethesda, MD, USA).

### 4.5. Enzyme Assays

The activities of NNT and G6PDH were assayed spectrophotometrically in cell homogenate (about 0.1 mg cell protein) using a Jasco double-beam/dual-wavelength spectrophotometer UV-550 (Jasco Inc., Easton, MD, USA).

The activity of NNT was assayed, essentially as described [6], in reverse using the analogue of NAD^+^, 3-acetylpyridine adenine dinucleotide (APAD), which has a higher oxidation potential than NAD^+^ and a different absorption spectrum especially for the reduced form [for refs see 6]. The reaction mixture was composed of 50 mM Tris (pH 8.0), 0.5% Brij-35, 1 mg/mL lysolecithin, 300 μM APAD and 300 μM β-NADPH. The reaction was initiated by the addition of 50 μg homogenate protein and the change in absorbance monitored at 375 nm for 3 min.

The activity of G6PDH was assayed as reported by Favia et al. [24].

### 4.6. Polarographic Measurements

O_2_ consumption was measured polarographically using a Gilson 5/6 oxygraph with a Clark electrode (Gilson Medical Electronics Inc., Middletown, WI) as in [22].

### 4.7. Assessment of Cellular and Mitochondrial ROS Production

Superoxide anion radical (O_2_^−•^) was detected both according to the adrenochrome method and by using the MitoSox dye [22,24], in order to distinguish specific mitochondrial ROS production from that occurring from other sources. In the adrenochrome method, the increase of absorbance at 480 nm, i.e., the conversion of epinephrine (no color) into adrenochrom (pink) with 1:1 stoichiometry, is the result of superoxide formation and was obtained by adding 0.5 mg protein of homogenate to 2 mL of PBS in the presence of epinephrine (1 mM). Absorbance increase was measured using a PerkinElmer lambda-5 spectrophotometer equipped with a thermostated holder. To specifically detect O_2_^−•^ production of mitochondrial origin, use was made of MitoSOX Red, a specific mitochondrial dye—highly and exclusively sensitive to superoxide [33], but not to other reactive oxygen/nitrogen species—which is selectively targeted to mitochondria where it accumulates as a function of mitochondrial membrane potential and exhibits fluorescence upon oxidation by superoxide and subsequent binding to mitochondrial DNA. Cell homogenate (0.1 mg protein/mL) in PBS was preincubated with succinate (10 mM) plus ADP (2.5 mM) for 2 min at 25 °C, so that it was a necessary energy source for the success of the procedure. After addition of 1 μM MitoSOX red, either in the absence or presence of different compounds (see legend to Figure 4), and further 15 min incubation, fluorescence emission at 580 nm was measured using a Perkin-Elmer LS-50B Luminescence Spectrofluorimeter (Perkin-Elmer Applied Biosystems, Foster City, CA).

### 4.8. NADPH and NADH Measurements

Nicotinamide adenine dinucleotide levels were measured by using the method involving 3-(4,5-dimethylthiazolyl-2)-2,5-diphenyltetrazolium bromide as terminal electron acceptor, as in [24,25,34].

### 4.9. Statistical Analysis and Computing

Biochemical data were expressed as means ± standard deviation of the mean and were representative of at least three separate experiments (*n* = independent experiments). Statistical significance of the data was evaluated using the one-way analysis of variance (ANOVA) followed by post-hoc Bonferroni test. Statistical differences for *p < 0.05.*

CorelDRAW Graphics Suite 11 program was used to create the artworks.

## 5. Conclusions

The overt oxidative stress condition observed in CF cell [23] elicits the loss of cell redox balance with deleterious consequences for metabolic regulation. Recently, we hypothesized that the inoperability of mitochondria paradoxically benefits the CF cell by lowering airway surface liquid glucose and ROS levels [23,24].

The findings of this study also support the hypothesis that reduced mitochondrial functionality may be an advantageous element since it finely regulates redox homeostasis, thus avoiding an announced bioenergetic catastrophe.

Notably, with the aim of shedding light on the metabolic environment of cell in CF and using the NNT inhibitor GSNO, the reverse mode of the NNT reaction is revealed, as occurs in human failing myocardium [6]. However, it is interesting to observe that in fact the direction of the NNT reaction is finely established by the cell’s energetic state, thus reminding us, as observed by Murphy [20], “that the direction of biochemical reactions in vivo is determined by local thermodynamics, not textbooks, potentially providing fresh insights into pathology and metabolic regulation.”

Now, in order to draw conclusions, if potentially the cell’s intention was to bolster the antioxidative capacity—as evidenced by the higher NNT protein level (Figure 1A)—to face up the oxidative load that advances, functionally in CF cells it happens that

(i) the cellular redox state, together with the low mΔΨ, pushes to drive NNT reverse reaction, at the expense of its antioxidant potential (Figure 2A,B);
(ii) working in reverse and under poor mitochondrial functionality conditions, the reduced activity of NNT prevents the NADH, produced by the reaction, from causing an explosion of ROS by the damaged respiratory chain (Figure 2A and Figure 4A) [22,24], as instead observed in the presence of (GLUT + ADP) (Figure 4A);
(iii) NNT activity reduction in the presence of 6AN, a G6PDH inhibitor, (Figure 3) endorses the crosstalk between different cellular compartments;
(iv) the greatly reduced level of mitochondrial ROS in NNT-loss cells suggests that the reverse NNT activity contributes substantially to their production (Figure 4A).
(v) The data are schematically reproduced in the picture (Figure 5). In particular, the mutual affection of NNT and G6PDH in the presence of the specific inhibitor of the other enzyme is clearly visible in the Figure: it represents a special condition supporting crosstalk between different cellular compartments.

To conclude, the findings of this study provide novel implications for the interconnectivity of bioenergetic pathways in CF. Unexpectedly, if on the one hand the dysregulated NNT activity should not cause ROS mitigation, as it loses its antioxidant capacity not producing NADPH, from the other, producing NADH, it allows its residual oxidation by Complex I—whose activity is reduced in CF but not fully blocked [25]—surely causing a low mitochondrial ROS production, but undoubtedly compromising flux through cytoplasmic NAD(P)H-requiring reactions. This data confirm what has already been proposed in previous works, namely that the reduction of mitochondrial respiration seems to be beneficial because it is responsible not only for reduction of glucose in the airway surface liquid [23,24], but also for the low level of ROS in CF cells (see Figure 4), thus suggesting that dysregulated NNT activity helps make cells right, contributing to define the overall pathologic phenotype of CF.

Anyway, although these interesting data were derived from human stabilized cell lines, representing one of the most widely used models to study CF pathology, we are aware that they will need to be further validated using additional cell models and mainly primary cells derived from CF patients. In this regard, increasing evidence indicates that primary airway epithelial cells provide the closest “in vitro” representation of the airway epithelium by providing a microenvironment or architecture closer to in vivo situations. Furthermore, in order to better mimic the primary tissue and surrounding microenvironment, primary cells are cultured in air–liquid interface conditions mimicking the in vivo situation, improving the clinical translational potential of the study.
